# Canadian pediatric eating disorder programs and virtual care during the COVID-19 pandemic: a mixed-methods approach to understanding clinicians’ perspectives

**DOI:** 10.1186/s12991-023-00443-4

**Published:** 2023-04-26

**Authors:** Kaylee Novack, Rachel Dufour, Louis Picard, Danielle Taddeo, Pierre-Olivier Nadeau, Debra K. Katzman, Linda Booij, Nicholas Chadi

**Affiliations:** 1 Department of Psychiatry and Addictology, Université de Montréal, Montréal, Canada; 2 Department of Psychology, Concordia University, Montreal, Canada; 3 Department of Psychology, Sainte-Justine University Hospital Centre, Montreal, Canada; 4 Division of Adolescent Medicine, Department of Pediatrics, Sainte-Justine University Hospital Centre, Université de Montréal, 3175 Chemin de la Côte-Ste-Catherine, Montreal, QC H3T 1C5 Canada; 5 Division of Adolescent Medicine, Department of Pediatrics, Hospital for Sick Children, University of Toronto, Toronto, Canada; 6 CHU Sainte-Justine Hospital Research Centre, Montreal, Canada; 7 Department of Psychiatry, Douglas Mental Health University Institute, McGill University, Montreal, Canada

**Keywords:** Pediatric, Eating disorders, COVID-19 pandemic, Virtual care, Healthcare professionals

## Abstract

**Background:**

As a result of the public health measures put in place during the COVID-19 pandemic in Canada, many health services, including those for the treatment of eating disorders, were provided at a distance. This study aims to describe the adaptations made in specialized pediatric eating disorder programs in Canada and the impact of these adaptations on health professionals’ experience of providing care.

**Methods:**

A mixed-methods design was used to survey healthcare professionals working in specialized pediatric eating disorder programs about adaptations to treatment made during the pandemic and the impact of these adaptations on their experience of providing care. Data were collected between October 2021 and March 2022 using a cross-sectional survey comprising 25 questions and via semi-structured interviews. Quantitative data were summarized using descriptive statistics and qualitative data were interpreted using qualitative content analysis.

**Results:**

Eighteen healthcare professionals in Canada completed the online survey, of whom six also participated in the semi-structured interviews. The cross-sectional survey confirmed that, unlike in pre-pandemic times, the majority of participants provided medical care (15/18) and mental health care (17/18) at a distance during the pandemic, with most participants using telephone (17/18) and videoconferencing (17/18). Most (16/18) health professionals indicated that virtual care would continue to be used as a tool in pediatric ED treatment after the pandemic. Participants used a combination of virtual and in-person care, with most reporting weighing patients both in clinic (16/18) and virtually (15/18). Qualitative content analysis generated five themes: (1) responding to increased demand with insufficient resources; (2) adapting to changes in care due to the COVID-19 pandemic; (3) dealing with uncertainty and apprehension; (4) virtual care as an acceptable and useful clinical tool, and (5) optimal conditions and future expectations. Most interview participants (5/6) had globally positive views of virtual care.

**Conclusions:**

Providing virtual multidisciplinary treatment for children and adolescents with eating disorders seemed feasible and acceptable to professionals during the pandemic. Moving forward, focusing on health professionals’ perspectives and providing appropriate training in virtual interventions is essential given their central role in successful implementation and continued use of virtual and hybrid care models.

**Supplementary Information:**

The online version contains supplementary material available at 10.1186/s12991-023-00443-4.

## Background

Eating disorders (EDs) are a group of mental illnesses that are mainly characterized by intense preoccupation with food, weight, and body image and are often associated with maladaptive eating behaviors, such as restriction and binging. These illnesses have serious physical and psychosocial health impacts on individuals [1] and, as a result, treatment generally requires a multidisciplinary care team. Further, hospitalization, day hospital care, and long-term follow-ups are often necessary when illness is more severe [2]. Effective treatment in the adolescent population is of particular concern as the peak onset of EDs occurs at this age [3].

During the COVID-19 pandemic, the severity and incidence of EDs were exacerbated [4]. Among children and adolescents in Canada, there was an increase in new ED presentations from a mean of 7.5 to 20.0 cases per month [5] and increases between 37 and 66% in emergency department visits and hospitalizations [6]. At the same time, many ED treatment programs for children and adolescents began providing virtual care out of necessity [7–11].

We will use the term virtual care to refer to any therapeutic approach delivered to individuals at a distance by computer, phone, or other technology modality, either synchronously or asynchronously. The approaches that have been most studied in ED treatment include asynchronous internet self-help, synchronous delivery of therapy via video or phone call (telemedicine), and the use of mobile applications.

In the adult population, internet self-help (especially programs based on Cognitive Behavioral Therapy) has been most robustly associated with a decrease in ED symptoms [12] although high-quality studies, using randomized-controlled designs, are limited [13, 14]. Less is known about the effectiveness of mobile phone apps and telemedicine [15, 16]. Nevertheless, a small number of studies carried out during the COVID-19 pandemic indicated that clinical effectiveness of telemedicine is similar to in-person care for EDs and comparable to historic benchmarks [11, 17–19] even if patients’ appreciation of these modalities is variable [4].

In the child and adolescent population, data regarding feasibility, acceptability, and effectiveness of all types of virtual care are limited [20]. Furthermore, perceived availability of virtual care in Canadian pediatric ED programs is currently unknown. The most recent data, from a 2010 survey aiming to characterize provincially recognized or hospital funded pediatric ED treatment programs in Canada, found that seven (64%) of the 11 identified sites reported using telemedicine to provide care to patients living at a significant distance from treatment centers [21]. In 2021, Canadian evidence-based guidelines for the use of all types of virtual care for EDs in the child and adolescent population were published [20]. Although these guidelines represent best practice, the state of current practice and the degree to which virtual care has been implemented in Canada remain unknown. In light of the increased demand for child and adolescent ED care in the context of the pandemic, we conducted a survey to characterize the use of virtual care among pediatric ED programs in Canada. This may serve to inform health professionals of standards in clinical practice.

## Methods

### Aims and hypotheses

The aim of this study was to describe the practices of pediatric ED programs in Canada with a focus on virtual care and adaptations made in the context of the COVID-19 pandemic. We also aimed to describe the healthcare professional experience in providing virtual care and adapted services.

We hypothesized that (1) all surveyed pediatric ED treatment programs integrated some form of virtual care into their services during the pandemic; and that (2) clinicians will have found virtual care to be an acceptable modality for pediatric ED treatment that can be retained and adapted in the future.

### Study design

We used a convergent parallel mixed-methods design for this study. Quantitative data from a cross-sectional survey were collected simultaneously to and supplemented by qualitative data from semi-structured interviews. Integration occurred at the analysis phase.

### Participants

#### Inclusion criteria

Participants had to be healthcare providers (nurses, psychologists, physicians, etc.) working at a publicly funded, specialized program that treats pediatric patients with EDs in Canada both prior to the onset of the COVID-19 pandemic (March 2019 to March 2020) and in the year after (April 2020 to the time of participation in the study). Targeted emails were sent to professionals in pre-identified programs located in tertiary pediatric hospitals. In Canada, there are 13 tertiary care pediatric hospitals each of which have designated inpatient eating disorder beds for adolescents. Community, general, and psychiatric hospitals may also hospitalize adolescents for eating disorders with varying levels of services and resources. Sites were chosen such that responses could be collected from at least one participant per province, although there were three provinces in which our team had no direct contacts. Potential participants were also reached via different Canadian Pediatric Society emailing lists (including the Adolescent Health section and the Mental Health Task Force). All invitations to participants contained an information sheet with a brief description of the study objectives and protocol in addition to the names and contact information of the principal investigators (LB and NC). We decided a priori that more than one participant from the same treatment program would be allowed to participate in the present study so that a broad multidisciplinary perspective on the research question could be captured.

#### Exclusion criteria

Participants were excluded if they did not provide responses to survey questions about care both prior to and during the pandemic given the study’s aim of comparing program characteristics at these 2 time points.

#### Selection for interview

Participants for the semi-structured interview were self-selected from among those health professionals who completed the online questionnaire. A question at the end of the survey prompted participants to provide their email address if they agreed to be contacted for the interview part of the study.

### Measures

#### Sociodemographic data

Gender, profession, and years of practice were collected from all online survey participants. No other sociodemographic information was collected to protect the anonymity of interview participants, given the small population of health professionals working in pediatric ED treatment programs in Canada.

#### Cross-sectional survey

Quantitative data were collected using an online survey that was hosted on the secure Qualtrics platform and composed of 25 questions (20 multiple-choice questions, 4 close-ended short answer questions, and 1 open-ended question). Topics covered in the survey included the volume of patients and services provided before and during the COVID-19 pandemic, the use of virtual care before and during the COVID-19 pandemic, the impact of the pandemic on specific aspects of treatment, and expectations for post-COVID adaptations to treatment. Since no existing questionnaire was available, questions were developed through discussions between members of a multidisciplinary research team, composed of three pediatricians specialized in adolescent medicine (NC, DT, DK), a child and adolescent psychiatrist (PON), two graduate students in psychiatric sciences and clinical psychology (KN, RD), and two clinical psychologists specialized in ED care (LP, LB). Content of the questions aimed to address emerging issues in the ED literature during the COVID-19 pandemic, that is (1) the increased demand for treatment [22, 23] and (2) the transition of services from in-person to virtual modalities [7, 8, 10, 24]. Thus, our questions sought to explore changes in patient volumes and the types of virtual care used specifically within the Canadian pediatric ED context. Questions and results from a previously published survey about pediatric ED programs across Canada [21] also served to inform content. Item writing was further guided by the recommendations of Johnson & Morgan [25]. A pilot version of the survey was tested by four pediatricians specialized in adolescent medicine and one psychologist to ensure clarity of the questions. Finally, the questionnaire was translated through a back translation procedure and was made available in both French and English. The English version of the quantitative survey is available in Additional file 1.

#### Interview

The interview guide was created through discussion between the multidisciplinary research team described above and based on relevant literature [7–10, 22, 24, 26]. The guide included five close-ended questions that gave participants the opportunity to describe the care provided at their center prior to and during the COVID-19 pandemic. The guide also included nine open-ended questions that explored health professionals’ personal experiences providing care during the pandemic, with particular attention paid to their appreciation and evaluation of virtual care. The guide was pilot tested by two physicians and one psychologist working in specialized pediatric ED programs and was translated to French using a back translation procedure. Interviews were conducted virtually and were audio and video recorded. These recordings were later used to transcribe the interviews verbatim. The English version of the interview guide is available in Additional file 2.

### Data analysis plan

#### Quantitative analyses

Given the small sample size, only descriptive analyses were conducted. Frequency measures, total counts, percentages, and proportions were used to describe and summarize the quantitative results of the survey. All participant responses were considered individually during analysis and there was no grouping of participants based on program sites. While this may skew results, it allowed us to protect participant anonymity, which was of particular concern in this study given the small number of health professionals working in specialized pediatric ED programs in Canada.

#### Qualitative analyses

Qualitative content analysis [27, 28] was chosen because of its flexible framework, which was appropriate for the exploratory nature of the study. The analysis was carried out as described by Bengtsson [28]. Transcripts were read multiple times and a code dictionary was subsequently established using deductive reasoning. All manuscripts were then coded such that the text could be cut and read by content category. Each coded unit was summarized—if necessary—and reorganized according to the broad concepts discussed. From here, themes were elaborated and refined iteratively until they were internally homogeneous and externally heterogeneous [27]. Results, summarizing each of the themes, were drafted. Direct quotations were not included in order to protect anonymity of participants. First author KN led the analysis under the supervision of senior author NC. Themes and final results were discussed by all team members. NVivo 12.7.0 was used to facilitate analysis.

### Researcher characteristics

The interviews were carried out by KN and RD under the supervision of co-PI NC. All have experience, clinically and/or in research, working in the field of pediatric EDs which allowed them to interpret the data with an informed perspective.

### Criteria of scientific credibility for qualitative analysis

Credibility was ensured through discussion between KN and co-investigators RD, LB, and NC during the analysis. Dependability was ensured by maintaining detailed records of all stages of the analysis and by validating the methodology with research team members. Confirmability was ensured by justifying methodological and analytical choices and by discussing and acknowledging potential biases.

### Ethics approval and consent to participate

The Scientific Committee of the Sainte-Justine University Hospital Center Ethics Committee reviewed and approved the study protocol. Participants provided electronic consent for the online questionnaire and verbal consent for the semi-structured interviews. In order to ensure participants’ confidentiality, survey and interview data did not contain any identifying information related to their workplace. Additionally, identifying information used for the purpose of contacting participants was kept separate from research data in a secure online server.

## Results

### Participants

The survey took place between October 26, 2021, and February 3, 2022. Overall, the survey link was opened on 67 occasions with 40 of these yielding no responses. Of the 27 responses, one was excluded as the participant did not work in a publicly funded, specialized program and 8 were excluded as they were incomplete—i.e., did not contain responses to questions about care both prior to and during the pandemic. Thus, 18 individuals were retained in the study sample. Fourteen participants (78%) were female. Participants included eight pediatricians (44.4%), four nurses (22.2%), two psychiatrists (11.1%), one nutritionist (5.6%), one therapist (5.6%), one psychologist (5.6%), and one social worker (5.6%). On average, participants had practiced for 19.2 years. Six of the health professionals who responded to the quantitative questionnaire (including three nurses, one social worker, one pediatrician, and one psychiatrist) subsequently participated in individual semi-structured interviews. On average, these interviews were 30–45 min in duration. They were conducted between December 3, 2021, and March 3, 2022.

### Program characteristics prior to the COVID-19 pandemic

Characteristics of the pediatric ED treatment programs (prior to the COVID-19 pandemic) where the 18 survey participants work are presented in Table 1.

**Table 1:** Participant-reported characteristics of eating disorder programs prior to the COVID-19 pandemic

Participant-reported program structure^1^	*n*
**Programs with inpatient units** (*N* = 18)	18
**Inpatient hospitalization**^2^ (*N* = 18)	
General psychiatric ward	7
General pediatric medicine ward	15
Specialized pediatric ED unit	8
Intensive care unit	1
Other	1
**Day treatment** (*N* = 18)	
Provided	11
**Day treatment structure**^2^ (*N* = 11)	
Full time	10
Part time	2
**Virtual care** (*N* = 18)	
Provided	7
**Patient volumes (monthly)**	*n*
**Inpatients** (*N* = 16)	
1–10	13
11–20	3
**New outpatient assessments** (*N* = 17)	
1–5	8
6–10	9
**Outpatient follow-ups** (*N* = 15)	
1–100	12
101–200	3
**Day program** (*N* = 11)	
1–5	6
6–10	5

^1^Data are reported at the participant level. There is a possibility that more than one participant reported data from the same eating disorder program

^2^Selection of more than one answer permitted

### Changes in patient volumes and characteristics during the COVID-19 pandemic

During the COVID-19 pandemic, increases in patient volumes in the inpatient and outpatient setting were reported by 83% (*n* = 15) and 89% (*n* = 16) of participants, respectively. Changes in patient volumes in inpatient, outpatient, and day treatment settings during the COVID-19 pandemic are described in Table 2.

**Table 2:** Number of participants reporting perceived percent change in patient volume during COVID-19 compared to prior

Treatment context			
Reported percent change in patient volumes	Inpatient (*N* = 18) *n* (%)	Outpatient (*N* = 18) *n* (%)	Day treatment (*N* = 11) *n* (%)
0–50% increase	5 (28%)	5 (28%)	1 (6%)
50–100% increase	6 (33%)	7 (39%)	0
> 100% increase	4 (22%)	4 (22%)	0
0–50% decrease	1 (6%)	2 (11%)	5 (28%)
50–100% decrease	1	0	0
> 100% decrease	0	0	4 (22%)
No change	1 (6%)	0	1 (6%)

Patterns of change in consultation frequency according to diagnostic category are displayed in Table 3. Overall, no clear pattern of change in frequency of visits was reported according to patient age, sex, racial/ethnic minority status, rurality, or socioeconomic class (data not shown).

**Table 3:** Number of participants reporting frequency of patient populations visits during COVID-19 compared to prior

Patient population					
	Anorexia nervosa (*N* = 17) *n* (%)	Bulimia nervosa (*N* = 17) *n* (%)	Avoidant/restrictive food intake disorder (*N* = 17) *n* (%)	Binge-eating disorder (*N* = 17) *n* (%)	Other specified feeding and eating disorder (*N* = 17) *n* (%)
More frequently	13 (76%)	9 (53%)	7 (41%)	4 (24%)	10 (59%)
Less Frequently	0	2 (12%)	3 (18%)	3 (18%)	0
No difference	4 (24%)	6 (35%)	7 (41%)	10 (59%)	7 (41%)

### Services offered in-person and virtually prior to and during the COVID-19 pandemic

The number of participants working in programs offering services virtually, in-person, and in day treatment prior to and during the COVID-19 pandemic is displayed in Table 4. The proportion of participants reporting working in programs offering virtual care more than doubled between the pre-pandemic (*n* = 7, 38.9%) and pandemic (*n* = 18, 100%) periods.

**Table 4:** Number of participants reporting medical/psychosocial services offered in-person and virtually prior to and during COVID-19

Type of services	In-person outpatient services	Day treatment		Virtual care	
	Pre-COVID^a^ (*N* = 17) *n* (%)	Pre-COVID (*N* = 11) *n* (%)	During COVID (*N* = 11) *n* (%)	Pre-COVID (*N* = 7) *n* (%)	During COVID (*N* = 18) *n* (%)
Medical care	15 (88%)	11 (100%)	6 (55%)	1 (14%)	16 (89%)
Nursing care	12 (71%)	11 (100%)	7 (64%)	3 (43%)	14 (78%)
Nutritional care	15 (88%)	11 (100%)	7 (64%)	6 (86%)	17 (94%)
Psychoeducation	12 (71%)	11 (100%)	7 (64%)	5 (71%)	15 (83%)
Family-based treatment (FBT)	15 (88%)	5 (45%)	5 (45%)	5 (71%)	13 (72%)
Individual therapy	12 (71%)	5 (45%)	5 (45%)	4 (57%)	12 (67%)
Group therapy	3 (18%)	11 (100%)	6 (55%)	0	7 (39%)
Meal support	2 (12%)	11 (100%)	7 (64%)	1 (14%)	11 (61%)
School	3 (18%)	10 (91%)	5 (45%)	0	2 (11%)
Art or music therapy	1 (6%)	8 (73%)	5 (45%)	0	0
Other^b^	5 (29%)	10 (91%)	3 (27%)	0	3 (17%)

^a^Data were missing from 1 participant regarding in-person outpatient services prior to the COVID-19 pandemic

^b^Other includes parent education groups, family therapy (not FBT), individual family support, occupational therapy, recreation therapy, body image group, coping skills group, cooking groups, mindfulness, self-discovery group, healthy lifestyles group, nutrition group, community building group, weekend planning and goal setting group, and yoga

Participants who reported not using virtual care prior to the COVID-19 pandemic (*n* = 11) were asked to identify all obstacles that prevented them from doing so. Most (*n* = 10, 91%) reported never having considered or discussed it. Lack of evidence to support implementation (*n* = 3, 27%), lack of financial and material resources (*n* = 3, 27%), and lack of training/trained providers (*n* = 2, 18%) were also endorsed.

### Modalities of virtual care used prior to and during the COVID-19 pandemic

With regard to the modalities of virtual care used, there was a 143% increase in the use of videoconferencing (*n* = 7 to *n* = 17) and a 325% increase in the use of telephones (*n* = 4 to *n* = 17) as modalities during the COVID-19 pandemic (see Table 5).

**Table 5:** Number of participants reporting use of virtual care modalities prior to, during, and after COVID-19

Modalities	Pre-COVID-19 pandemic (*N* = 18) *n* (%)	During COVID-19 pandemic (*N* = 18) *n* (%)	Expected to be permanent^a^ (*N* = 17) *n* (%)
Videoconferencing^b^	7 (39%)	17 (94%)	15 (88%)
Telephone^c^	4 (22%)	17 (94%)	9 (53%)
Virtual day program	0	2 (11%)	1 (6%)
Virtual meal support	1 (6%)	11 (61%)	4 (24%)
Texting	0	1 (6%)	1 (6%)
Apps	2 (11%)	1 (6%)	1 (6%)
Other^d^	1 (6%)	0	0

^a^Data were missing from 1 participant regarding modalities of virtual care expected to be permanent

^b^Videoconferencing refers to the use of a videoconferencing platform for at least one of the following including: appointments with a medical provider, appointments with a mental health or behavioral health provider, interdisciplinary care appointments, case conferences, inpatient or outpatient care rounds, individual therapy, family therapy, training providers outside of the participant’s treatment program, and parent education groups

^c^Telephone refers to the use of telephone (voice call) for appointments with a medical provider, appointments with a mental health or behavioral health provider, interdisciplinary care appointments, and training providers outside of the participant’s treatment program

^d^Other includes telehealth assessments or appointments with community teams

Participants used a number of methods for weighing patients during the pandemic, including weighing patients in the clinic (*n* = 16, 89%), having patients weighed at home by a caregiver (*n* = 15, 83%) and not weighing patients at all (*n* = 3, 17%). With regard to the frequency of physical examinations during the pandemic, 56% of participants (*n* = 10) reported a decrease, 39% (*n* = 7) reported no change, and one participant reported an increase.

A summary of open-ended responses to the question “what lessons did you and your team learn during the COVID-19 pandemic that will change how your program provides ED treatment in the future?” is provided in Additional file 3.

### Qualitative results

Qualitative content analysis of the verbatim transcripts generated 5 main themes: (1) responding to increased demands with insufficient resources; (2) adapting to changes in care due to the COVID-19 pandemic; (3) dealing with uncertainty and apprehension; (4) virtual care as an acceptable and useful clinical tool; and (5) optimal conditions and future expectations. Each of these is discussed in more detail below.

#### Responding to increased demand with insufficient resources

Almost all participants spoke about perceived increases in patient volumes as the biggest challenge they faced during the pandemic, using words such as “astronomical” and “exponential” to describe the change. Most participants’ programs did not have additional resources to deal with the increase in demand. As a result, the clinical workloads of professionals increased significantly during the pandemic. Given this increase in patient volumes, participants described the difficulty of having to triage effectively and “getting the most urgent ones in faster” while balancing regular follow-ups and clinical responsibilities. This was a major stressor for many healthcare professionals. One participant pointed out that providing high-quality care across all of their different practices (e.g., outpatient, inpatient, and specialized clinics) was overwhelming because referrals and admissions for patients with EDs were so much higher than prior to the pandemic.

#### Adapting to changes in care due to the COVID-19 pandemic

Participants described having to make numerous changes to their care programs during the COVID-19 pandemic. Most notably, participants described new additions to care, which consisted of virtual care and, for one participant, the creation of a home hospitalization program. For all participants, virtual care meant the provision of treatment at a distance using a videoconferencing platform such as Zoom, Teams, or others. Overall, half of the participants’ programs transitioned completely to virtual care at the start of the pandemic. One participant’s program offered optional virtual care and two participants’ programs continued to provide almost entirely in-person care as usual, with occasional use of telephone appointments. However, as the pandemic progressed, a number of participants made changes to these initial adaptations in order to better suit the needs of ED patients and their specific contexts. This included transitions (1) from a completely virtual model to a hybrid model, with weight, height, and vital sign collection in-person and a multidisciplinary appointment online; (2) from a completely virtual model to a combination of virtual and in-person appointments, in which medically unstable patients were prioritized for in-person care, and (3) from a completely in-person model to alternating virtual medical appointments and in-person nursing and nutritional appointments (during which weight, height, and vital signs were monitored). Two participants mentioned that staff received specific training prior to implementation of virtual care, with one of them still describing the transition as somewhat challenging. Virtual care also opened new possibilities for providing large-scale training on EDs, with one participant describing a provincial virtual education session on EDs for nurse practitioners and a provincial family-based treatment training program. Virtual groups (including yoga and multi-family FBT) were mentioned as being well received by patients.

In addition to implementing virtual care, other changes in care mentioned by participants were limitations in physical space for therapeutic activities, reductions in staffing capacity and patient census, and cancellations of programming, appointments, and in one case an entire day program.

#### Dealing with uncertainty and apprehension

Almost all the participants interviewed in this study had no experience using virtual care prior to COVID-19 pandemic. Many participants seemed worried about using a new modality. Several participants echoed that they found the transition difficult or that they initially were not convinced that virtual care would be useful. Two participants who had used Telehealth, which was not well received by patients, presupposed that virtual care would be similarly disliked. There was apprehension about the unknown impacts that virtual care might have on patients. There were also concerns about the logistical hurdles that had to be overcome in setting up virtual family meals and weighing sessions. It seemed that hesitancy towards using virtual care was more common among those with less experience using technology. Interestingly, those participants who used virtual care less throughout the pandemic lacked confidence in using it and remained uncertain about its usefulness at the time of the study while health professionals who used it regularly because they were obligated tended to gain more confidence and appreciate it more over time.

#### Virtual care as an acceptable and useful clinical tool

During the interviews, health professionals discussed both advantages and disadvantages to the use of virtual care for treating youth with EDs (see Table 6). However, five of the six participants expressed globally positive views of virtual care and were open to its continued use.

**Table 6:** Perceived advantages and disadvantages of virtual care from the perspective of healthcare professionals

Perceived advantages	Perceived disadvantages
Increased flexibility to meet patient, family, and provider needs Increased accessibility to care Perceived as a viable alternative to in-person care No change in therapist–patient relationship and establishment of therapeutic alliance Facilitated team organization and collaboration	Inequity in access to technology for providers and patients/families Privacy concerns for patients in their homes Technological difficulties Logistical and administrative difficulties Difficulty establishing therapeutic alliance and engaging unmotivated patients Difficulty reconciling ED care principles with limitations of virtual care (e.g., allowing patient to have a scale in their home to allow for weighing at a distance) Work-from-home and virtual meetings impede informal collaboration between colleagues Patients logging into sessions in public places Body image concerns may be exacerbated for patients when seeing themselves on screen

While no participants felt that virtual care could completely replace in-person care, many agreed it is a helpful, additional tool that will be used to increase access to care for out-of-town patients, to make care more flexible for patients and families, and to provide group therapy, education, and programming (Table 6).

#### Optimal conditions and future expectations

Participants discussed what they believed would be optimal conditions for the use of virtual care in the future based on their experiences throughout the pandemic. Participants believed virtual care is most effective when users have reliable technology, guidelines and education for use, and rapid access to IT services. Strong leaders who promote virtual care among their teams were also important to participants. Additionally, the best care seems to be provided when confidentiality is clearly discussed and when patients are motivated and have family support. Half of the participants noted that meeting with patients and establishing a relationship prior to virtual care is helpful.

Participants also reflected on their future expectations within the field of pediatric EDs. Four participants agreed that they would continue to deal with the increased demand for services, with many of them seemingly overwhelmed by this thought. As a result of this continued demand, participants spoke about how it will be necessary to find ways to increase capacity. All expected virtual care to continue and to potentially expand to new modalities.

## Discussion

The current study examined practices and perspectives of healthcare professionals working in pediatric ED programs in Canada during the COVID-19 pandemic relative to pre-pandemic times. Most surveyed participants did not previously use virtual care, however, the majority were able to transition a large portion of their services to be delivered virtually and all participants reported that their program had incorporated some form of virtual care during the pandemic. There was an increase in the use of videoconferencing and telephone as modalities for care during the pandemic. Medical, nursing, and nutritional care were most commonly provided virtually but there were also increases in virtual group therapy and FBT. Programs combined virtual modalities with elements of in-person care that are crucial in ED follow-ups, such as measurement of weight, height, and vital signs. The quantitative survey showed a perceived increase in patient volumes across inpatient and outpatient settings and a perceived decrease in patient volumes in day programs.

Content analysis of qualitative data from the semi-structured interviews complemented the quantitative data by adding the personal perspective of six healthcare professionals. Participants stressed that the biggest challenge they faced was responding to the surge in demand for pediatric ED services. Ultimately, the stress of triaging patients, stretching insufficient human and material resources, and ensuring quality care for those who most urgently needed it was the backdrop against which professionals also made the transition towards providing virtual care. In the context of these challenges and adaptations, participants reported experiencing uncertainty and apprehension. Despite this, participants generally viewed virtual care in a positive light, weighing the flexibility and accessibility it affords patients, families, and professionals more strongly than its disadvantages. Participants suggested that virtual care seems to represent a viable tool that has and will maintain its place alongside usual care in specialized pediatric ED programs. Looking to the future, optimal conditions for virtual care were suggested.

The increase in demand for services across inpatient and outpatient settings described in this study was high, with approximately one third of participants reporting 50–100% increases and one-fifth reporting greater than 100% increases. This was consistent with studies conducted in Canada [5, 6] and elsewhere [4, 22, 29] during the first year of the pandemic.

Despite high uptake of virtual care overall, few participants used virtual day treatment programs. Though this could be attributed to the higher complexity of adapting day treatment programs to virtual modalities, it should be noted that others have reported on the feasibility of virtual day treatment during the pandemic [29, 30]. Hybrid models of care, combining in-person and virtual elements, were prevalent, however, among our participants. This may be due to the high risk for medical instability among patients and, thus, the need to physically monitor patients at close intervals. Thus, there is potential to expand various models of care within pediatric ED treatment programs in Canada.

Participants in our study, while initially apprehensive, nonetheless had generally favorable impressions of virtual care overall. Though some studies of professionals working in ED treatment show similarly positive attitudes [29, 31], other studies, especially those carried out early in the pandemic, reported that only a minority of users had positive experiences with virtual care [32, 33]. Perceptions in the literature are, thus, mixed. Discrepancies in perception may be influenced by the timing of these studies—i.e., early in the pandemic as compared with later—because clinicians may have become more comfortable with virtual care as time went on. It is important that studies carried out during the pandemic be understood within the context of the exceptional public health measures and the isolation individuals may have been experiencing at the time. Preference for treatment modalities should continue to be studied outside of strict lockdown periods when the use of virtual care is not imposed on health professionals.

Acknowledging health professionals’ attitudes towards virtual care is important as they are key actors in its successful implementation. Health professionals are more likely to accept changes they are prepared for [34, 35], that they value, and that will likely lead to direct patient benefits [35]. Initial attitudes towards virtual care may be especially important because mental health professionals who endorsed lower perceived quality of online therapeutic relations and had more negative attitudes towards online therapy and its efficacy before having used it also eventually reported more obstacles during its use [36]. Similarly, perceived positive potential of online therapy predicted future intention to use it [37]. So, even if participants in this study and others [10, 32] tended to describe a decrease in uncertainty and apprehension over the course of the pandemic, these findings have important implications for the future. Offering training in virtual care from the outset may facilitate use and uptake.

There are several strengths to the current study. The combination of both quantitative and qualitative data allowed for a broad overview of health professional’s experience with virtual care and the provision of ED treatment during the COVID-19 pandemic. The involvement of a multidisciplinary research team—including physicians, clinical psychologists, and graduate students—contributed to the development of a protocol, questionnaire, and interview guide that are specific and clinically relevant both to the area of focus and the study population. Finally, the multidisciplinary perspective of the research team reduced the risk of bias during qualitative data analysis.

There are some limitations to consider when interpreting our findings. Most notably, the generalizability of these results should be considered cautiously owing to the small sample size and the anonymous nature of the sample. As we did not ask participants to report the name of the program where they work, we could not ensure that participants were representative of all health professionals across Canada nor could we adjust for responses that may have been submitted by multiple participants from a single site, which could skew results. Further, the small sample and the format of the questions only allowed for descriptive statistics and visual representations of the data, meaning relations between variables could not be examined. While our decision to include diverse health professionals in the study may have introduced heterogeneity into the sample, quantitative outcomes, which focused on practices common to all clinicians rather than ones particular to one field, should have been little impacted. Furthermore, the inclusion of diverse viewpoints was a strength in the qualitative section of the study, which sought to gather perspectives of a broad range of clinicians working in pediatric ED programs in Canada. Finally, the questionnaire that was developed for this study was not validated, and, thus, its psychometric properties remain unknown. To counter this, questions were piloted in a small sample similar to the population of interest to ensure they were appropriate and feasible. Given these limitations, results should not be seen as an average but as a representation of the individual realities of the health professionals who participated in the study.

## Conclusion

The implementation of virtual care within pediatric ED programs across Canada during the COVID-19 pandemic is generally acceptable to health professionals, many of whom agreed it is a viable therapeutic modality. Participants implemented virtual care into their programs in a variety of ways, according to their needs and contexts. Hybrid models were prevalent and can offer the advantages of both in-person and virtual care. Participants expect that the large increase in patient volumes that began during the pandemic will likely continue to be problematic and strategic integration of virtual care will be helpful. Given that the majority of participants expect virtual care will become a standard part of pediatric ED treatment, promoting positive attitudes toward it—e.g., through training programs—may ease implementation. Questions remain regarding effectiveness of virtual care for the treatment of EDs in the pediatric population. Randomized controlled trials are necessary to address this but given the widespread use of virtual care nationally, large-scale naturalistic studies may be an important next logical step to study the usefulness of virtual care in the real-world setting. Nevertheless, future studies and training programs should continue to focus on health professionals’ perspective given their central role in successful implementation of virtual or hybrid models of care.

## Supplementary Information

**Additional file 1:** Quantitative Cross-Sectional Questionnaire

**Additional file 2:** Semi-Structured Interview Guide

**Additional file 3:** Lessons learned by pediatric ED health professionals during the COVID-19

## Data Availability

The datasets generated and analyzed during the current study are not publicly available to protect the anonymity of participants but may be available from the corresponding author on reasonable request.

## Abbreviations

ED: Eating disorder
FBT: Family-based treatment
