# Supplementary material for: Canadian pediatric eating disorder programs and virtual care during the COVID-19 pandemic: a mixed-methods approach to understanding clinicians’ perspectives
Source: Ann Gen Psychiatry. 2023 Apr 26;22:16. doi: 10.1186/s12991-023-00443-4 (PMC10132795; doi:10.1186/s12991-023-00443-4)
Supplement: Supplementary file 1 — Additional file 1: Quantitative Cross-Sectional Questionnaire [file 12991_2023_443_MOESM1_ESM.docx]

Additional File 1: Quantitative Cross-Sectional Questionnaire

National Survey of Eating Disorders Treatment Programs

**Care Prior to the COVID-19 Pandemic**
The following questions refer to care provided to children and adolescents with eating disorders in **your eating disorder program before the COVID-19 pandemic.**
 
This refers to the period from **March 2019 to March 2020.**

**Outpatient clinic (excluding any form of day program/day hospital care)**

|  |
| --- |

1. How many initial consultations were typically done in your outpatient clinic **each month?**

________________________________________________________________

|  |
| --- |

2. How many follow-up medical visits were typically done in your outpatient clinic **each month**?

________________________________________________________________

3. What services did your program provide to outpatients? Please check all that apply.

- Medical care
- Nursing care
- Nutritional care/counselling
- Meal support
- Individual psychotherapy. Please specify the type of therapy or therapeutic approach: __________________________________________________
- Psychoeducation
- Art or music therapy
- School services
- Family-based treatment (e.g., Maudsley approach)
- Group therapy. Please specify the type of therapy or therapeutic approach: __________________________________________________
- Other (e.g., yoga, occupational therapy, etc.). Please specify the type of service offered: __________________________________________________

| Page Break |  |
| --- | --- |

**Inpatient care**

The following questions refer to care provided to children and adolescents with eating disorders in **your eating disorder program before the COVID-19 pandemic.**
 
This refers to the period from **March 2019 to March 2020**.

4. Did you admit pediatric patients with eating disorders to your facility for inpatient care?

- Yes
- No

5. Where were the patients typically hospitalized? Please check all that apply.

- General pediatric/medical inpatient unit
- General psychiatry inpatient unit
- Specialized pediatric eating disorder unit
- Intensive care unit
- Other. Please specify: __________________________________________________

6. Excluding day treatment, how many patients were typically hospitalized for eating disorders **per month** in your facility?

________________________________________________________________

7. What services did your program provide for hospitalized patients? Please check all that apply.

- Medical care
- Nursing care
- Nutritional care/counselling
- Meal support
- Individual psychotherapy. Please specify the type of therapy or therapeutic approach: __________________________________________________
- Psychoeducation
- Art or music therapy
- School services
- Family-based treatment (e.g., Maudsley approach)
- Group therapy. Please specify the type of therapy or therapeutic approach: __________________________________________________
- Other. (e.g., yoga, occupational therapy, etc.). Please specify the type of service offered: __________________________________________________

| Page Break |  |
| --- | --- |

**Day program/day hospital care**

The following questions refer to care provided to children and adolescents with eating disorders in **your eating disorder program before the COVID-19 pandemic.**
 
This refers to the period from **March 2019 to March 2020**.

8. Did your program offer either a day program or day hospital care, e.g., a program offering care or services several hours or days per week beyond weekly outpatient clinic visits?

- Yes
- No

|  |
| --- |

9. Please provide the number of patients that were typically admitted to day program or day hospital care **per month.**

________________________________________________________________

10. Please define the structure of your day program or day hospital care by checking all that apply.

- Full-time (5 days per week)
- Part-time (e.g., some days per week or half-days)
- Hourly basis (some hours per week)
- Other structure. Please specify: __________________________________________________

11. Which of the following services did your program provide as part of the day program or day hospital care? Please check all that apply.

- Medical care
- Nursing care
- Nutritional care/counselling
- Meal support
- Individual psychotherapy. Please specify the type of therapy or therapeutic approach: __________________________________________________
- Psychoeducation
- Art or music therapy
- School services
- Family-based treatment (e.g., Maudsley approach)
- Group therapy. Please specify the type of therapy or therapeutic approach: __________________________________________________
- Other. (e.g., yoga, occupational therapy, etc.). Please specify the type of service offered: __________________________________________________

End of Block: Care prior to the COVID-19 pandemic

Start of Block: Virtual Care

**Virtual Care**
For the purpose of this survey, **virtual care** is defined as any interaction between patients and/or families and/or members of their circle of care occurring remotely, including, but not limited to: any services delivered through short-message service (SMS), synchronous tools (e.g., scheduled and 'on demand' videoconferences), asynchronous tools (e.g., emails), and remote home-monitoring (e.g., smartphone or app-based).
 
The following questions refer to care provided to children and adolescents with eating disorders in **your eating disorder program before the COVID-19 pandemic.**
 
This refers to the period from **March 2019 to March 2020.**

12. Was virtual care a part of eating disorder treatment in your program?

- Yes
- No

13. What were the main reasons that virtual care was not a part of eating disorder treatment in your program? Please check all that apply.

- Lack of training/trained providers
- Lack of financial/material resources
- Lack of evidence to support implementation
- Never considered/discussed
- Other. Please specify: __________________________________________________

14. What modalities of virtual care were used in your program? Please check all that apply.

- Video conferencing for appointments with a medical provider
- Video conferencing for appointments with a mental health/behavioural health provider
- Video conferencing for interdisciplinary care/appointments
- Video conferencing for case conferences
- Video conferencing for inpatient or outpatient care rounds
- Telephone (voice call) for appointments with a medical advisor
- Telephone (voice call) for appointments with a mental health/behavioural health provider
- Telephone (voice call) for interdisciplinary care/appointments
- Video conferencing for individual therapy
- Video conferencing for family therapy
- Virtual day program/day hospital care
- Virtual meal support
- Text messaging for patient care
- Use of smartphone applications for tracking patient progress
- Video conferencing for training providers outside of your program/organization
- Telephone (voice call) for training providers outside of your program/organization
- Other. Please specify: __________________________________________________

15. What services were offered using virtual care modalities, either partially or completely? Please check all that apply.

- Medical care
- Nursing care
- Nutritional care/counselling
- Meal support
- Individual psychotherapy. Please specify the type of therapy or therapeutic approach: __________________________________________________
- Psychoeducation
- Art or music therapy
- School services
- Family-based treatment (e.g., Maudsley approach)
- Group therapy. Please specify the type of therapy or therapeutic approach: __________________________________________________
- Other. (e.g., yoga, occupational therapy, etc.). Please specify the type of service offered: __________________________________________________

16. Were any of the following virtual care modalities suggested for patients and their families? Please check all that apply.

- Virtual care provided by outside/community providers
- Smartphone/self-help applications
- Educational websites
- Online training/education modules
- Discussion forums
- Other. Please specify: __________________________________________________
- None of the above were offered

End of Block: Virtual Care

Start of Block: Care during the COVID-19 pandemic

**Care during the COVID-19 Pandemic**
The following questions refer to care provided to children and adolescents with eating disorders in **your eating disorder program during the COVID-19 pandemic.** 
 
The "COVID-19 pandemic" refers to the period **from April 2020 to June 2021**. 

17. Did your program experience a change in patient volumes with regards to:

|  | No change | 0-25% increase | 25-50% increase | 50-100% increase | >100% increase | 0-25% decrease | 25-50% decrease | 50-100% decrease | >100% decrease |
| --- | --- | --- | --- | --- | --- | --- | --- | --- | --- |
| New outpatient assessments |  |  |  |  |  |  |  |  |  |
| Inpatient care |  |  |  |  |  |  |  |  |  |
| Day program and day hospital patients |  |  |  |  |  |  |  |  |  |

| Page Break |  |
| --- | --- |

The following questions refer to care provided to children and adolescents with eating disorders in **your eating disorder program** **during the COVID-19 pandemic.**
 
The "COVID-19 pandemic" refers to the period from **April 2020 to June 2021.**

18. What modalities of virtual care, if any, did your program use during the COVID-19 pandemic? Please check all that apply.

- No virtual care provided
- Video conferencing for appointments with a medical provider
- Video conferencing for appointments with a mental health/behavioural health provider
- Video conferencing for interdisciplinary care/appointments
- Video conferencing for case conferences
- Video conferencing for inpatient or outpatient care rounds
- Telephone (voice call) for appointments with a medical provider
- Telephone (voice call) for appointments with a mental health/behavioural health provider
- Telephone (voice call) for interdisciplinary care/appointments
- Video conferencing for individual therapy
- Video conferencing for family therapy
- Virtual day program/day hospital care
- Virtual meal support
- Text messaging for patient care
- Use of smartphone applications for tracking patient progress
- Video conferencing for training providers outside of your program/organization
- Telephone (voice call) for training providers outside of your program/organization
- Other (please specify) __________________________________________________

19. What services did your program provide virtually during the COVID-19 pandemic? Please check all that apply.

- Medical care
- Nursing care
- Nutritional care/counselling
- Meal support
- Individual psychotherapy. Please specify the type of therapy or therapeutic approach: __________________________________________________
- Psychoeducation
- Art or music therapy
- School services
- Family-based treatment (e.g., Maudsley approach)
- Group therapy. Please specify the type of therapy or therapeutic approach: __________________________________________________
- Other. (e.g., yoga, occupational therapy, etc.) Please specify the type of service offered: __________________________________________________
- No virtual care provided

20. Which of the following services did you offer as part of your **day program or day hospital care** during the COVID-19 pandemic? Please check all that apply.

- Day-program or day-hospital was closed during COVID-19 pandemic
- Medical care
- Nursing care
- Nutritional care/counselling
- Meal support
- Individual psychotherapy. Please specify the type of therapy or therapeutic approach: __________________________________________________
- Psychoeducation
- Art or music therapy
- School services
- Family-based treatment (e.g., Maudsley approach)
- Group therapy. Please specify the type of therapy or therapeutic approach: __________________________________________________
- Other. (e.g., yoga, occupational therapy, etc.) Please specify the type of service offered: __________________________________________________

21. How did the COVID-19 pandemic affect methods for weighing patients? Please check all that apply.

- Patients were weighed in the clinic in a similar manner to before the onset of the pandemic
- Patients were weighed in the clinic in a different manner to before the pandemic. Please specify. __________________________________________________
- Patients were weighed at home by a caregiver.
- Patients were not weighed
- Other: __________________________________________________

22. How did the COVID-19 pandemic change the frequency at which physical examination was completed at regular medical follow-up appointments?

- Increased frequency of physical examination
- Decreased frequency of physical examination
- No change in frequency of physical examination

23. During the COVID-19 pandemic, were some populations seen more or less frequently in your program, as compared to before the pandemic?

|  | No difference | More frequently | Less frequently |
| --- | --- | --- | --- |
| Patients with anorexia nervosa |  |  |  |
| Patients with bulimia nervosa |  |  |  |
| Patients with ARFID |  |  |  |
| Patients with binge eating disorder |  |  |  |
| Patients with OSFED |  |  |  |
| Patients < 14 years old |  |  |  |
| Patients 14 years or older |  |  |  |
| Male patients |  |  |  |
| Female patients |  |  |  |
| Patients from racial and/or ethnic minorities |  |  |  |
| Patients from rural areas |  |  |  |
| Patients from urban areas |  |  |  |
| Patients of low socioeconomic status |  |  |  |
| Patients of high socioeconomic status |  |  |  |
| Other group: |  |  |  |

End of Block: Care during the COVID-19 pandemic

Start of Block: Lessons learned

**Lessons Learned from the COVID-19 Pandemic**

24. What adaptations made to ED treatment at your program during the COVID-19 pandemic do you anticipate **will be permanent**? Please check all that apply.

- Video conferencing for appointments with a medical provider
- Video conferencing for appointments with a mental health/behavioural health provider
- Video conferencing for interdisciplinary care/appointments
- Video conferencing for case conferences
- Video conferencing for inpatient or outpatient care rounds
- Telephone (voice call) for appointments with a medical provider
- Telephone (voice call) for appointments with a mental health/behavioural health provider
- Telephone (voice call) for interdisciplinary care/appointments
- Video conferencing for individual therapy
- Video conferencing for family therapy
- Virtual day program/day hospital care
- Virtual meal support
- Text messaging for patient care
- Use of smartphone applications for tracking patient progress
- Video conferencing for training providers outside of your program/organization
- Telephone (voice call) for training providers outside of your program/organization
- Other (please specify) __________________________________________________
- No adaptations made during the COVID-19 pandemic will be permanent

25. What lessons did you and your team learn during the COVID-19 pandemic that will change how your program provides eating disorder treatment in the future?

________________________________________________________________

________________________________________________________________

________________________________________________________________

________________________________________________________________

________________________________________________________________

End of Block: Lessons learned

Start of Block: Participant demographics

**Participant information**

Gender:

- Male
- Female
- Non-binary
- Other: __________________________________________________

Profession:

- Pediatrician
- Psychiatrist
- Nurse
- Psychologist
- Social worker
- Psycho-educator/counsellor
- Nutritionist
- Other (please specify): __________________________________________________

|  |
| --- |

Number of years in practice:

________________________________________________________________

End of Block: Participant demographics

Start of Block: Thank you

We thank you for taking the time to respond to this survey. 

End of Block: Thank you
