# Supplementary material for: Canadian pediatric eating disorder programs and virtual care during the COVID-19 pandemic: a mixed-methods approach to understanding clinicians’ perspectives
Source: Ann Gen Psychiatry. 2023 Apr 26;22:16. doi: 10.1186/s12991-023-00443-4 (PMC10132795; doi:10.1186/s12991-023-00443-4)
Supplement: Supplementary file 2 — Additional file 2: Semi-Structured Interview Guide [file 12991_2023_443_MOESM2_ESM.docx]

Additional File 2: Semi-Structured Interview Guide

**Qualitative Interview Guide**

**Qualitative interview on current practices in eating disorder treatment for children and adolescents across specialized care programs in Canada.**

**Introduction:**

Thank you for agreeing to participate in a video-chat interview today. My name is (insert name) and, as a reminder, we are meeting today to discuss a bit more about your and your clinic’s experience of pediatric care for eating disorders, including the challenges and lessons that were learned from the COVID-19 pandemic.

**Informed consent - Confirm email consent and ask for questions:**

*This part should be read verbatim.*

Before today, you received and signed via email a consent form detailing the parameters of this study, including this interview. I will go over the highlights from that email again now, as I want to ensure you’re comfortable and do not feel obliged to participate in any way.

Following our interview today, your data will be saved in a secure location and your identity as well as your institution’s identity will be kept confidential. Research assistants, including myself, will transcribe what is said here today from the audio recordings, and those recordings will then be destroyed.

The questions I will ask today will be about your team's experiences with care for eating disorders patients before and during the COVID-19 pandemic, with a focus on what adaptations were made and how virtual care was used/will be used in the future.

You are free to withdraw from this discussion at any time with no negative consequences. If, after we finish today, you decide you are not comfortable with some of the things you said, please contact me within one week, and I will take these statements out of the transcript.

Do you have any questions about your rights as a participant in this interview?

In the future, if you have any questions about your rights as a research participant, you can refer to the consent form.

**Interview Questions:**

1. Could you list the specific diagnoses as well as the severity criteria for eligibility in your eating disorder program? __________________________________________________________________________________________________________________________________________
2. If available at your center, could you describe your day treatment/day hospital program, including admission criteria, capacity, and care provided before the COVID-19 pandemic?

__________________________________________________________________________________________________________________________________________

1. Before the beginning of the COVID-19 pandemic, what virtual care modalities were offered in your program? Can you provide a brief description of the services offered? Were they offered in the context of a study? __________________________________________________________________________________________________________________________________________
   1. Potential prompts: For example, was telemedicine/tele-practice being used in the past? Were there group sessions or discussions with the family taking place over the phone or over video chat?
   2. Potential prompts: What was the frequency/duration of appointments, the providers/services involved, how were virtual treatments combined with in-person treatment?
2. Since the beginning of the COVID-19 pandemic, what adaptations, if any, were made to the structure of the care provided at your center?

__________________________________________________________________________________________________________________________________________

- 1. Potential prompts: With regards to inpatient care? Outpatient assessments (new patients)? Outpatient follow-ups? Day-hospital / day program treatment?

1. Since the beginning of the COVID-19 pandemic, what adaptations, if any, were made with regards to the professionals involved in providing care to ED patients?

__________________________________________________________________________________________________________________________________________

- 1. Potential prompts: For example, those working in inpatient care? Outpatient care? Day hospital/day treatment program? Was it possible to provide the same nutritional, social work, psychological support as usual?

1. What was your experience adapting to the changes mentioned above?

__________________________________________________________________________________________________________________________________________

- 1. Potential prompts: Were the changes hard to implement across the clinic? Were some of the adaptations more beneficial than others? Would you have done anything differently?

1. What would you say are some of the strengths and weaknesses of the adaptations you mentioned earlier?

_______________________________________________________________________________________________________________________________________________________________________________________________________________

1. What would you say were the biggest challenges your clinic faced during the pandemic?

_______________________________________________________________________________________________________________________________________________________________________________________________________________

- 1. Potential prompts: Did your clinic experience a surge in new consultations? Were there technical difficulties involved in transitioning to virtual care? Was it difficult to maintain therapeutic alliances virtually? Was it difficult to maintain contact with patients?

1. When it comes to virtual care specifically, what do you think are the optimal conditions for it to be effective with ED patients?

__________________________________________________________________________________________________________________________________________

1. What do you think are the disadvantages of virtual care and/or what makes its use complicated?

__________________________________________________________________________________________________________________________________________

- 1. Potential prompts: Are there administrative challenges? Is technology available for providers, parents and patients?

1. Based on your experiences, can you comment on:
   1. Your perception of provider appreciation of virtual care (including your own and your team’s)?

______________________________________________________________________________________________________________________________

- - 1. Potential prompt: Were there impact on clinical care? On communication among team members? On teaching opportunities?
  1. Your perception of patient/family appreciation of virtual care?

______________________________________________________________________________________________________________________________

- - 1. Potential prompts: Do you think they would be open to continuing treatment virtually, either partially or completely? Did you receive any comments or feedback, positive or negative, from them?
  1. The impacts of virtual care on collaboration with external providers

________________________________________________________________________________________________________________________

- - 1. Potential prompts: Were there impacts on teaching/training opportunities? Communication with external providers?

1. What adaptations made to ED treatment at your program during the COVID-19 pandemic do you anticipate will be temporary?

__________________________________________________________________________________________________________________________________________

1. What adaptations made to ED treatment at your center during the COVID-19 pandemic do you anticipate will be permanent?

__________________________________________________________________________________________________________________________________________

1. Do you have any additional comments about:
   1. Your ED treatment program, in particular? _______________________________________________________________
   2. The COVID-19 pandemic period in the context of EDs? _______________________________________________________________
   3. Anticipated challenges and/or opportunities for the future in the context of EDs? _______________________________________________________________

Thank you for taking the time to participate in this interview.
