# Supplementary material for: Canadian pediatric eating disorder programs and virtual care during the COVID-19 pandemic: a mixed-methods approach to understanding clinicians’ perspectives
Source: Ann Gen Psychiatry. 2023 Apr 26;22:16. doi: 10.1186/s12991-023-00443-4 (PMC10132795; doi:10.1186/s12991-023-00443-4)
Supplement: Supplementary file 3 — Additional file 3: Lessons learned by pediatric ED health professionals during the COVID-19 [file 12991_2023_443_MOESM3_ESM.docx]

Additional File 3: Lessons learned by pediatric ED health professionals during the COVID-19

What lessons did you and your team learn during the pandemic that will change how your program provides ED treatment in the future?

| Theme | Sub-theme (number of mentions) |
| --- | --- |
| Advantages of virtual care | Increased accessibility for rural patients (n=1) and overall (n=2)  The flexibility of providing both in-person and virtual care makes treatment easier for families (n=1)  Virtual care can help to keep patients in their communities and improve skills of local care teams (n=1)  Providing care to patients virtually in their home environment’ increases clinician understanding (n=1) |
| Disadvantages of virtual care | Lack of privacy at home (n=1)  Virtual meetings have direct negative impacts on team building and feeling of connection between staff; support for new staff is lacking (n=1)  Virtual team meetings are less focused due to clinician multitasking (n=1) |
| Identification of organizational strategies for virtual care implementation | Hybrid model is preferred over 100% virtual (n=3)  Video is superior to telephone for engaging patients (n=1)  Virtual care is easier and more helpful for individual as opposed to family appointments (n=1)  New triage system implemented to distinguish between general mental health and specific ED care needs for patients with EDs (n=1)  The cancellation of temporary leave from the hospital (i.e., so that patients can visit home on the weekend) helped hospitalized patients gain weight more quickly during their inpatient treatment (n=1) |
| Identification of needs for pediatric ED clinics in Canada | Training is needed for rural providers (n=1)  Need to increase regional/provincial capacity for treatment to meet increased demand for care (n=1)  Need to set clear expectations for patients and families with regards to behavior during virtual appointments (n=1) |
| Overall perception of virtual care | Virtual care is feasible (n=2) and acceptable but has limitations.  Virtual care is efficient and effective (n=1)  Virtual care is very impactful (n=1)  Will continue to use virtual care (n=2) for FBT in particular (n=1)  Virtual care was destigmatized; the rapid transition accelerated its uptake (n=1)  Despite rapid transition to virtual care, did not learn how to cope with increase in patient referrals (n = 1)  Virtual care is only useful for those who cannot access in-person care because of distance (n=1)  Virtual care works poorly with adolescent patients (n=1) |
